# Supplementary material for: Development of a cognitive bias methodology for measuring low mood in chimpanzees
Source: PeerJ. 2015 Jun 11;3:e998. doi: 10.7717/peerj.998 (PMC4465942; doi:10.7717/peerj.998)
Supplement: Supplemental Information 1 [file peerj-03-998-s001.pdf]

Bateson & Nettle

READ ME file for Chimp_data_MS.csv

The accompanying .csv file contains the following variables:

Date: the date on which the data was collected

Time: the time of day the data was collected: am (A) or pm (P)

Chimp: the name of the chimpanzee (Bobby, ET or Nicky)

Session: the session number (integer: 1-n)

Phase: the phase of training/testing. Six-trial blocks (BT), three-trial blocks (H), choice trials (C) or test (T)

Trial: the trial number (integer: 1-n)

Valence: the valence of the cone presented (in forced trials) or chosen (in choice trials). Positive (P), near positive (NP), mid (M), near negative (NP) or negative (N).

Side: the side on which the cone was presented (in forced trials) or of the cone chosen (in choice trials). Right (R) or left (L).

Latency: the latency in seconds to touch the cone (forced trials) or the first cone (choice trials).

Carrot: Whether or not a piece of carrot was presented, and if so whether the chimp touched it before the cone (1), after the cone (2) or not at all within 60 s (0).
